# Is saltmarsh restoration success constrained by matching natural environments or altered succession? A test using niche models

**DOI:** 10.1111/1365-2664.13033

**Published:** 2017-11-22

**Authors:** Martin J. P. Sullivan, Anthony J. Davy, Alastair Grant, Hannah L. Mossman

**Affiliations:** ^1^ School of Geography University of Leeds Leeds UK; ^2^ School of Biological Sciences University of East Anglia Norwich UK; ^3^ School of Environmental Sciences University of East Anglia Norwich UK; ^4^ School of Science and the Environment Manchester Metropolitan University Manchester UK

**Keywords:** de‐embankment, habitat restoration, managed realignment, niche models, plant community, redox, saltmarsh, species distribution model, species occurrence, tidal frame

## Abstract

1. Restored habitats, such as saltmarsh created through managed realignment, sometimes fail to meet targets for biological equivalence with natural reference sites. Understanding why this happens is important in order to improve restoration outcomes.
2. Elevation in the tidal frame and sediment redox potential are major controls on the distribution of saltmarsh plants. We use niche models to characterize 10 species’ responses to these, and test whether differences in species occurrence between restored and natural saltmarshes in the UK result from failure to recreate adequate environmental conditions.
3. Six species occurred less frequently in recently restored marshes than natural marshes. Failure of restored marshes to achieve the elevation and redox conditions of natural marshes partially explained the underrepresentation of five of these species, but did not explain patterns of occurrence on older (>50 years) restored marshes.
4. For all species, an effect of marsh age remained after controlling for differences in environmental conditions. This could be due to differences in successional mechanism between restored and natural marshes. In recently restored marshes, high‐marsh species occurred lower in the tidal frame and low‐marsh species occurred higher in the tidal frame than in natural marshes. This supports the hypothesis that competition is initially weaker in restored marshes, because of the availability of bare sediment across the whole tidal frame. Species that establish outside their normal realized niche, such as *Atriplex portulacoides*, may inhibit subsequent colonization of other species that occurred less frequently than expected on older restored marshes.
5. *Synthesis and applications*. Niche models can be used to test whether abiotic differences between restored sites and their natural counterparts are responsible for discrepancies in species occurrence. In saltmarshes, simply replicating environmental conditions will not result in equivalent species occurrence.

## Abstract 2

Niche models can be used to test whether abiotic differences between restored sites and their natural counterparts are responsible for discrepancies in species occurrence. In saltmarshes, simply replicating environmental conditions will not result in equivalent species occurrence.

## 1 INTRODUCTION

The creation of new habitat through restoration is an important strategy to compensate for loss or degradation of natural and semi‐natural ecosystems (Young, 2000). Habitat restoration has been attempted in a wide range of systems and locations, but restoration success is variable (Suding, 2011), and a predictive understanding of why restoration success varies is needed (Brudvig, 2017). Such variation is influenced by the environmental suitability of restored sites and other filters on initial community assembly, and subsequent successional development, yet the relative importance and interaction of these has rarely been studied (Brudvig et al., 2017). Niche models have been widely used to aid conservation management, for example by predicting species distributions under climate change (Araújo, Cabeza, Thuiller, Hannah, & Williams, 2004), but their potential to predict restoration outcomes, and the mechanisms leading to these, has yet to be realized (Brudvig, 2017; Brudvig et al., 2017). Here, we apply niche models to test whether environmental suitability accounts for outcomes in restored saltmarsh, and to test for signatures of different successional mechanisms.

Saltmarsh is often restored through managed realignment (MR), where sea defences are breached to reinstate tidal inundation onto previously reclaimed land. The saltmarsh seedbank has been lost during the period of embankment (Erfanzadeh, Garbutt, Pétillon, Maelfait, & Hoffmann, 2010) and, in the UK, no seeding or planting is carried out, so colonization depends on “natural” dynamics. Other breaches to sea defences occurred during historic storm surges, allowing us to study the long‐term (50–100 years) development of “restored” saltmarsh. Saltmarsh restoration is subject to stringent targets to compensate for losses due to development and coastal erosion (European Commission, 2000). However, the plant communities that develop on restored marshes are not equivalent to those on natural marshes (Mossman, Davy, & Grant, 2012a; Wolters, Garbutt, & Bakker, 2005b), even after many decades (Garbutt & Wolters, 2008), with underrepresentation of characteristic mid‐marsh species such as *Plantago maritima*,* Limonium vulgare* and *Triglochin maritima* (Mossman et al., 2012a).

These discrepancies may stem from environmental conditions after restoration not matching those on natural marshes. One of the main determinants of saltmarsh plant distribution is elevation in the tidal frame (Davy, Brown, Mossman, & Grant, 2011). MR often occurs on low‐lying agricultural land (French, 2006) and low elevation could lead to the dominance of pioneer species (Garbutt, Reading, Wolters, Gray, & Rothery, 2006). Plant occurrence is also influenced by sediment redox potential (Davy et al., 2011), which is correlated with elevation and would be expected to be lower in restored marshes, but is even lower in restored marshes than at equivalent elevations in natural marshes (Mossman et al., 2012a). Nevertheless, some restored sites are high in the tidal frame or have oxic sediments (Brooks, Mossman, Chitty, & Grant, 2015; Mossman et al., 2012a), so differences in environmental conditions may not fully account for the discrepancies. If they do, future restoration schemes should seek to manipulate topography prior to flooding so that elevation and redox conditions more closely match those in natural marshes.

Alternatively, differences in propagule availability, reduced competition and subsequent priority effects could mean that restored sites undergo different trajectories of succession, even where environmental conditions match those in natural marshes (Young, Petersen, & Clary, 2005). Natural saltmarshes develop by facilitated succession, where initial submergence‐tolerant colonists increase sedimentation, raising the level of the marsh surface and facilitating the growth of less tolerant species (Bertness & Shumway, 1993; Castellanos, Figueroa, & Davy, 1994). A wider range of conditions may be available in restored marshes and so facilitated succession may not be needed to create suitable conditions for high‐marsh species (Hughes, Fletcher, & Hardy, 2009). This would reduce the importance of competition, as species could colonize bare sediment wherever conditions are suitable; while under facilitated succession, species must invade existing lower marsh vegetation. Patch‐scale experimental studies have demonstrated the role of competition in setting upper and lower elevation limits of saltmarsh plants (Hacker & Bertness, 1999; Pennings, Grant, & Bertness, 2005), so we predict that reduced competition would allow pioneer species to grow higher in the tidal frame, and upper‐marsh species lower in the tidal frame. Such shifts in realized niche on restored marshes could have longer term consequences for vegetation development. Upper‐marsh species with high dispersal ability may be able to colonize bare sediment at mid‐elevations and hinder the establishment of more dispersal‐limited species, resembling the inhibition model of succession (Connell & Slatyer, 1977). Then, management would need to focus on helping dispersal‐limited species overcome these priority effects.

We examine plant communities from 22 saltmarshes including natural (reference), MRs (<10 years post breach) and older accidently restored marshes (ARs, >50 years post breach), and use niche modelling to examine (1) whether differences in elevation and redox potential are sufficient to explain differences in species distribution between restored and natural marshes and (2) whether any shifts in niche position between natural marshes and MRs are consistent with the expected signatures of altered successional processes.

## 2 MATERIALS AND METHODS

### 2.1 Study sites

Saltmarshes were sampled in three regions of the UK; Essex, Norfolk and the Humber Estuary. In each region, MR sites were selected based on criteria of age and accessibility at time of sampling. Nearby natural reference sites and, where available, accidental realignments (AR) were also selected. Reference sites were selected for size (>2 ha), proximity to at least one realigned site and accessibility. The age of reference marshes is not known. The maximum distance between MR and nearest natural marsh was 1.8 km. In total, 22 sites were sampled, of which 7 were MRs, 4 were ARs and 11 were natural marshes (NAT) (Table S1). There was no current or known historical grazing by livestock on any site.

### 2.2 Field survey

Sites were sampled once between 2004 and 2010, between July and October. Restored sites were sampled in the same year as their nearest natural marsh. The percentage cover and occurrence (presence/absence) of all plant species and bare ground was assessed in 0.5 m × 0.5 m quadrats. We used percentage cover to select the species for inclusion in the study (see below) and occurrence data in the niche models. Quadrats were located along at least two transects, where each transect spanned the elevation range of each site and started at a random location along the shore. At least 30 quadrats were surveyed at each site, with the number of quadrats proportional to the area of the site and to the distance from the upper strandline to mudflats in each site. In total, 290 quadrats were surveyed in MRs, 249 in ARs and 506 in natural marshes.

Redox potential was measured at 5 cm depth in the centre of each quadrat, using a BDH (British Drug Houses, Lutterworth, UK) Gelplas combination redox electrode with an Ag/AgCl reference, standardized with respect to a standard hydrogen electrode (+204 mV). The elevation above Ordnance Datum Newlyn (ODN) of the centre of each quadrat was measured using a differential GPS (Topcon, Newbury, UK) with vertical accuracy and precision of <2 cm. Elevation was standardized as relative tidal height (RTH) based on in situ measurements of local tidal regime (Mossman, Davy, & Grant, 2012b) or data recorded at nearby ports, so that locations with the same RTH have the same duration of submergence and emergence. RTH was calculated as RTH = Elevation relative to ODN−MHWN/(MHWS−MHWN), where MHWN is the level of mean high water neap tide and MHWS is the level of mean high water spring tide. RTH = 0 at MHWN and 1 at MHWS.

### 2.3 Study species

We investigated the environmental associations of 10 species: *Spartina anglica*,* Salicornia* spp., *Suaeda maritima*,* Aster tripolium*,* Puccinellia maritima*,* Atriplex portulacoides*,* L. vulgare*,* T. maritima*,* Plantago maritima* and *Elytrigia atherica*. Nomenclature follows Stace (2010). They were the 10 most abundant species recorded, comprising 89.9 ± 22.5% (*SD*) of vegetation cover at the sites. They also represented a range of ecological strategies (Table S2), including annual pioneers (e.g. *Suaeda*,* Salicornia*), potential dominants (e.g. *Puccinellia, Atriplex*,* Elytrigia*) and mid‐marsh species typically deficient in MRs and ARs (*Limonium*,* Triglochin*,* Plantago*; Mossman et al., 2012a). *Salicornia* was treated as *Salicornia europaea* agg. because of the uncertain status and difficulty in identification of the micro‐species (Davy, Bishop, & Costa, 2001).

### 2.4 Data analysis

All statistical analyses were carried out using r (R Core Team 2014). We examined whether redox potential and RTH varied among saltmarsh type and restoration age‐classes (i.e. MRs, ARs and natural marshes), hereafter “age,” by constructing linear models for each response variable with marsh age and region as explanatory factors. We then tested whether the relationship between redox and RTH varied with marsh age using a linear model with marsh age, RTH, region and the RTH–marsh age interaction as explanatory variables; a significant interaction would indicate a change in the form of the redox–RTH relationship.

We used Gaussian process (GP) niche models to relate the occurrence (presence/absence in a quadrat) of each plant species to environmental conditions. GP models were chosen as they can capture complex ecological responses to environmental variables, including nonlinear interactions among predictor variables, while avoiding the overfitted response surfaces that often result from machine learning techniques (Golding & Purse, 2016). The use of GP models for niche modelling is described by Golding and Purse (2016). Briefly, the probability of a species occurring in a given quadrat is considered to relate to an underlying latent variable that varies over environmental space (defined as the quadrat by explanatory variable matrix) following a multivariate Gaussian distribution. The probability of a species occurring in a quadrat is related to the latent Gaussian variable via a probit link. Inference over the latent variable is obtained in a Bayesian framework using Laplace approximation. We used the GRaF r package (Golding, 2014) to fit GP models, using default settings and a flat (i.e. uninformative) prior for the latent variable.

Separate GP models were constructed for each species. We first trained GP models using data from natural marshes, with redox potential, RTH and region as explanatory variables. The purpose the region term was to account for processes acting at a larger spatial scale than a quadrat, such as differences in seed availability or climate, which may influence the occurrence of species. We assessed the predictive performance of these models through 1,000 cross‐validation runs, randomly selecting 75% of data points for model training and using the remaining 25% of data points to test model performance. We used the area under the receiver operating characteristic curve (AUC) as a measure of model ability to discriminate between presences and absences. AUC is sensitive to the relative occurrence area of species, leading to a tendency for models of species found in a small number of samples to have higher AUC values (Lobo, Jiménez‐Valverde, & Real, 2008), so the proportion of quadrats a species is recorded in should be considered when interpreting values (see Table S3).

To test whether the occurrence of species is expected to vary between different‐aged marshes based on the availability of environmental conditions alone, we used the natural marsh trained models to predict the probability of occurrence of each species in quadrats in MRs and ARs. To propagate uncertainty from the GP model into this derived analysis, we obtained 1,000 predictions from the model's posterior distribution. For each set of predictions, we calculated the mean occurrence probability across quadrats for each marsh age, and present the median and 95% CIs for these means.

To assess the effect of marsh age on species occurrence while controlling environmental variation, we fitted a second GP model for each species, with redox potential, RTH, region and marsh age class as explanatory variables. We then predicted the occurrence of each species in each marsh age, setting other explanatory variables to the values that give the maximum value of the latent variable, and present the posterior mode and 95% credible intervals for these occurrence probabilities. If our models capture the environmental determinants of a species’ distribution, then these predictions can be interpreted as the probability of a species occurring in a quadrat in each marsh age given suitable environmental conditions. In practice, this interpretation will be approximate as unmodelled environmental variables will affect species distributions.

We used Kruskal–Wallis tests to examine whether species’ position in the tidal frame (RTH in quadrats where species present) differed among marsh age categories, with pairwise Mann–Whitney–Wilcoxon tests applied post hoc. To examine whether changes in position in the tidal frame differed from expectations if species were selecting the most similar environmental conditions to those occupied on natural marshes, we used the natural marsh trained GP models to predict the occurrence probability of each species in each quadrat in MRs and ARs. For each marsh age, we took the *n* quadrats with the highest occurrence probability, where *n* is the number of quadrats the species occurred in for that marsh age, and calculated the mean RTH. We repeated this for 1,000 samples of the posterior distribution of the GP model to account for uncertainty in the niche models.

## 3 RESULTS

### 3.1 Niches of saltmarsh plants on natural marshes

Three species, *Spartina, Salicornia* and *Suaeda*, occurred below MHWN, while all species were recorded above MHWS. The same three species occurred in quadrats with very low redox potentials (lower than −200 mV), but were also present in some of the most oxic quadrats (Table S4). The range of RTHs occupied by species on natural marshes was negatively correlated with the median RTH they occurred at (*r* = −.80, *df* = 8, *p *= .005). Likewise, the range of redox potentials occupied by species was negatively correlated with the median redox potential in quadrats they occupied, although this was not statistically significant (*r* = −.57, *df* = 8, *p* = .082). Thus, species occurring in the low marsh tend to occur in a broader range of environmental conditions than those higher in the marsh.

**Figure 1:**
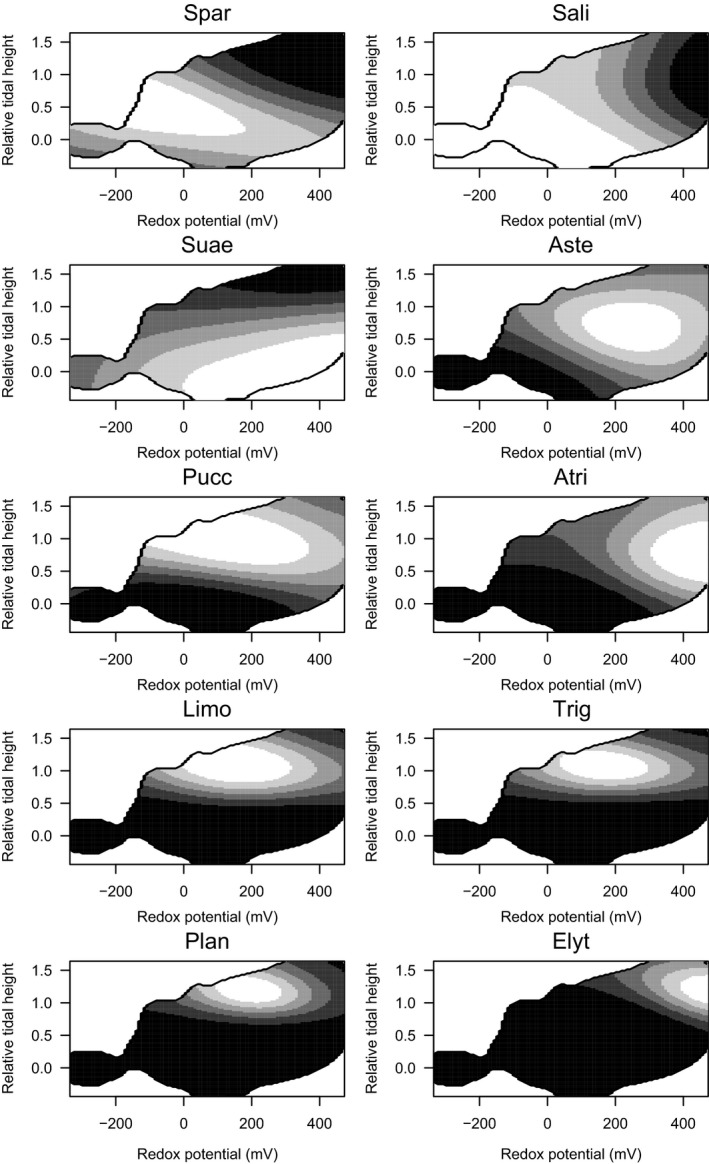
Modelled niches of 10 plant species in natural saltmarshes: *Spartina anglica* (Spar), *Salicornia europaea* agg. (Sali), *Suaeda martima* (Suae), *Aster tripolium* (Aste), *Puccinellia martima* (Pucc), *Atriplex portulacoides* (Atri), *Limonium vulgare* (Limo), *Triglochin maritima* (Trig), *Plantago maritima* (Plan) and *Elytrigia atherica* (Elyt). The occurrence of each species in quadrats in natural marshes was modelled as a function of redox potential, relative tidal height and region using Gaussian Process models. Niches have been clipped by available environmental conditions. Lighter shading indicates more suitable conditions

Despite the broad absolute tolerances of some species to RTH and redox (Table S4), species differed in their association with these variables (Figure 1, Table S5). *Salicornia* could occur in most of the environmental space sampled, but was most strongly associated with low in the tidal frame and low redox (Figure 1). *Spartina* was also associated with low redox potentials, but had a preference for higher in the tidal frame than *Salicornia*, while *Suaeda* was most likely to occur in quadrats that were low in the tidal frame but had relatively high redox potentials (Figure 1). Niche differences were also evident among mid‐marsh species, with *Atriplex* being most likely to occur in quadrats with high redox potentials while *Limonium*,* Triglochin* and *Plantago* were more likely to occur in quadrats at similar heights in the tidal frame but with lower redox potentials (Figure 1). *Elytrigia* was restricted to quadrats near or above MHWS and with high redox potential (Figure 1). The role of RTH and redox in determining plant distribution was supported by the ability of niche models to predict the species’ occurrence when tested using cross‐validation (mean AUC = 0.74, Table S3).

### 3.2 Differences in environmental conditions between restored and natural saltmarshes

**Figure 2:**
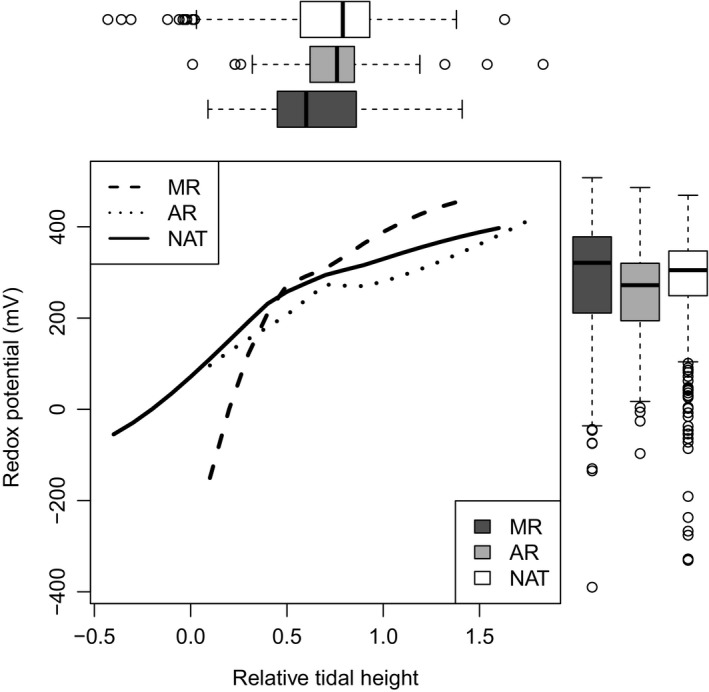
Environmental conditions in managed realignments (black, *n* = 289 quadrats), accidental realignments (grey, *n* = 249 quadrats) and natural (white, *n* = 506 quadrats) marshes. The relationship between relative tidal height and redox potential is shown by locally weighted polynomial regression fits

Elevation and redox conditions overlapped considerably across all marsh ages (Figure 2). Nevertheless, some differences in mean conditions were evident. MRs tended to be at lower elevations than natural marshes (*t* = 6.7, *p *< .0001, Figure 2), but elevations in ARs were not significantly different from those in natural marshes (*t* = 0.1, *p* = .890, Figure 2). In contrast, redox conditions were not significantly different in MRs and natural marshes (*t* = 1.6, *p *= .117, Figure 2), but were lower in ARs (*t *= 3.9, *p *= .0001, Figure 2). Redox potential was positively related to RTH in natural marshes (β = 238 ± 14.4, *t* = 16.6, *p *< .0001), with this relationship becoming steeper in MRs (*t *=* *2.2, *p *=* *.026) and less steep in ARs (*t *= 2.8, *p *= .0047, Figure 2).

### 3.3 Do differences in environmental conditions explain differences in plant occurrence?

**Figure 3:**
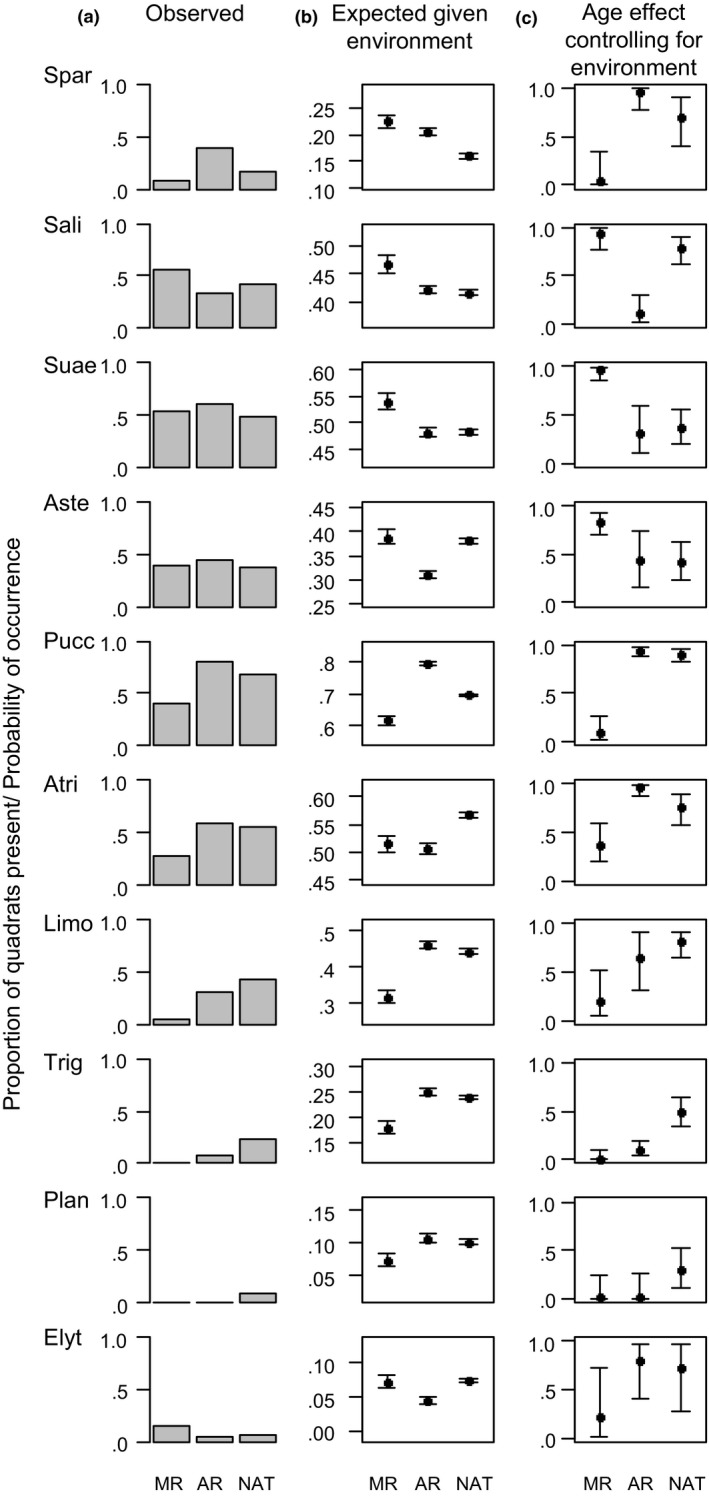
Occurrence of 10 plant species in managed realignments (MR), accidental realignments (AR) and natural saltmarshes (NAT). Species names are as in Figure 1. (a) Observed proportion of quadrats occupied in each marsh age class. (b) Predicted mean probability of occurrence across all quadrats in each marsh age class based on available environmental conditions. Predictions were made using niche models trained on natural marsh data. Error bars show 95% CIs based on 1,000 samples from the posterior distribution of the niche model. (c) Effect of marsh age controlling for differences in environmental conditions. The predicted probability of occurrence in each marsh age class when other environmental variables are at their most favourable are shown. Error bars show 95% credible intervals. For example, *Spartina* occurs less frequently in quadrats in MRs than in natural marshes (a), but is predicted to have a higher occurrence probability in MRs than natural marshes based on available environmental conditions (b). The probability of occurrence of *Spartina*, given suitable environmental conditions, is predicted to be lower on MRs than natural marshes (c)

Niche model predictions indicated that these differences in environmental conditions between natural and restored marshes would lead to differences in species occurrence (Figure 3). *Spartina*,* Puccinellia*,* Atriplex*,* Limonium, Triglochin* and *Plantago* were recorded less frequently in MRs than in natural marshes (Figure 3a). MRs were predicted to be less suitable than natural marshes for the latter five species based on available redox and RTH conditions, but were predicted to be more suitable for *Spartina* (Figure 3b). Marsh age was an important control on the occurrence of these species (Table S5), with a negative effect of MR, independent of environmental conditions, for all these species (Figure 3c). In contrast, *Salicornia*,* Suaeda* and *Aster* were more likely to occur on MRs than natural marshes given suitable environmental conditions (Figure 3c).

*Limonium*,* Triglochin* and *Plantago* all occurred less frequently in ARs than natural marshes (Figure 3a). However, the ARs were predicted to be similarly or more suitable for these species than natural marshes (Figure 3b), indicating that the availability of suitable elevation and redox conditions does not explain the lower frequency of these species on ARs. Instead, there was a negative effect of marsh age (Figure 3c), although 95% credible intervals for the probability of occurrence in ARs overlapped with those for the probability of occurrence in natural marshes for *Limonium* and *Plantago*. The role of marsh age was supported by the results of hierarchical partitioning, which showed that marsh age was the most important determinant of the occurrence of these species (Table S5). The potential dominants *Spartina*,* Puccinellia* and *Atriplex* occurred more frequently in ARs than in natural marshes (Figure 3a). For *Spartina* and *Puccinellia*, ARs were predicted to be more suitable than natural marshes, but ARs were predicted to be less suitable for *Atriplex* (Figure 3b). There was a weak positive effect of AR for all these species (although 95% credible intervals overlapped with those for natural marsh for all these species), with the strongest effect evident for *Atriplex* (Figure 3c).

### 3.4 Differences in species’ position in the tidal frame between restored and natural marshes

**Figure 4:**
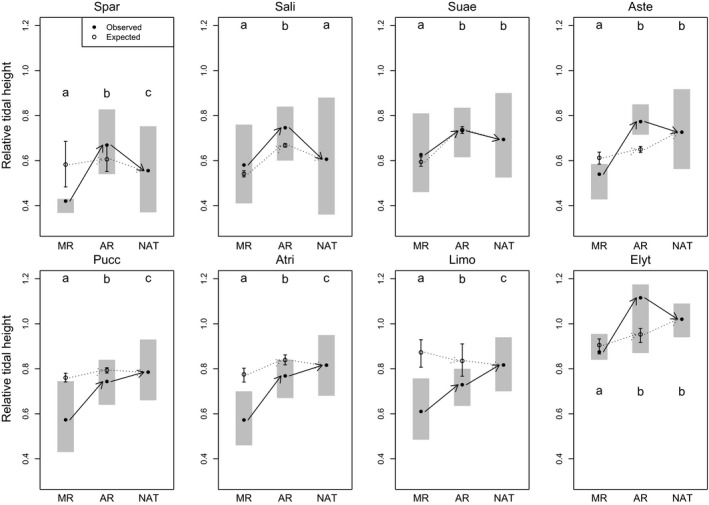
Change in distribution in the tidal frame (relative tidal height) of eight plant species with saltmarsh age (names as in Figure 1). Note that there were insufficient records of *Plantago* and *Triglochin* in managed realignments for this analysis. The mean relative tidal height of quadrats occupied by a species in managed realignments (MR), accidental realignments (AR) and natural marshes (NAT) are shown by filled black circles, with grey rectangles showing the interquartile range. Open circles show the expected mean elevation for each species if they were occupying the most suitable quadrats in MRs and ARs predicted by natural marsh trained niche models. Error bars show the 95% CI for this mean. Solid arrows show the trajectory of observed changes, dotted arrows show the trajectory of expected changes. Letters denote significant differences (*p *< .05) in observed tidal height associations between marsh ages based on pairwise Mann–Whitney–Wilcoxon tests with false discovery rate adjusted *p*‐values

The median RTHs at which species occurred differed significantly between marsh types (Kruskal–Wallis tests, χ^2^ ≥ 19.6, *p *< .001). However, differences in environmental conditions on MRs meant that many species would be expected to occur lower in the tidal frame in MRs based on their niches on natural marshes (Figure 4). Eight species occurred on average at lower tidal heights in MRs (*Triglochin* and *Plantago* had to be omitted from this analysis as they were too infrequent on MRs), although this difference was not statistically significant for *Salicornia* (Figure 4). The four mid‐ and upper‐marsh species (*Puccinellia*,* Atriplex*,* Limonium*,* Elytrigia*), as well as *Aster* (a mid‐marsh pioneer), occurred lower in the tidal frame in MRs than expected given available environmental conditions (although the difference was not significant for *Elytrigia*), while the low‐marsh pioneers *Salicornia* and *Suaeda* occurred higher in the tidal frame (Figure 4). Species’ positions in the tidal frame were more similar in ARs and natural marshes, although *Spartina* occurred higher and *Puccinellia, Atriplex* and *Limonium* occurred lower (Figure 4). For the latter three species, these differences were greater than expected given available environmental conditions, while *Salicornia, Aster* and *Elytrigia* occurred higher in the tidal frame than expected (Figure 4).

## 4 DISCUSSION

### 4.1 Controls on species distributions in natural marshes

The occurrence patterns of saltmarsh plants on natural marshes revealed by extensive field sampling and niche modelling are generally consistent with results of previous studies at single sites. Redox potential and elevation have important independent effects on the distribution of saltmarsh plants (Davy et al., 2011), and this is reflected in the high discriminatory power of niche models incorporating these variables. Local variations in redox potential are likely to be important in determining plant occurrence, as species with similar RTH associations differed in their redox associations (Figure 1), while the relative importance of redox varied among species (Table S5). Variation in redox at a particular tidal height may result from local topography. Low redox potential is associated with waterlogging, so is lower in local depressions and higher on well‐drained mounds (Castellanos et al., 1994; Varty & Zedler, 2008). Around MHWS, *Atriplex* was associated with high redox potential, as reported previously (Crooks, Schutten, Sheern, Pye, & Davy, 2002; Davy et al., 2011), while *Puccinellia*,* Limonium, Triglochin* and *Plantago* were associated with lower redox potential. Thus, local depressions in the mid‐ and upper‐marsh may increase species richness by favouring the latter species over *Atriplex* (Varty & Zedler, 2008).

### 4.2 Do environmental differences account for differences in species occurrence between natural and restored marshes?

Surface elevation was generally lower in MRs than in natural marshes, likely due to sediment compaction during agricultural land use and lack of sediment inputs during embankment (French, 2006), and redox increased more quickly with RTH compared to in natural marshes (Figure 2). Limited availability of low redox conditions at higher elevations may be disadvantageous to mid‐marsh species that favour relatively low redox conditions, such as *Triglochin* (Figure 3). These mid‐marsh species do occur less frequently on MRs than natural marshes, while low‐marsh pioneer species occur more frequently. However, these mid‐marsh species also occur less frequently on ARs (Mossman et al., 2012a), where, following decades of sediment input, the flatter relationship between RTH and redox meant that there was greater availability of low redox conditions high in the tidal frame. Indeed, *Puccinellia*, which had a similar niche to *Triglochin*,* Limonium* and *Plantago*, occurred more frequently in ARs than natural marshes. Availability of suitable low redox conditions in the mid‐ and upper‐marsh is therefore unlikely to explain the underrepresentation of these species on ARs. This is supported by presence of marsh age effects for these species in niche models controlling for RTH and redox (Figure 3).

### 4.3 Alternative explanations for differences in species occurrence

If differences in plant occurrence between restored and natural marshes result from differences in succession, we would expect these to leave signatures in the zonation of plant species distributions (Davy, 2000). We predicted that pioneer species typical of the low marsh would occur at higher elevations in restored marshes because they are not yet out‐competed by high‐marsh species (Bertness, 1991). Conversely, we predicted species typical of the high marsh to occur at lower elevations, because they do not need to be able to invade low‐marsh vegetation to colonize. Our data support both predictions. Pioneer species occurred at higher elevations in MRs than expected given available environmental conditions, in line with observations that *Salicornia* and *Suaeda* occurred at higher elevations on MR sites shortly after restoration (Hughes et al., 2009). Mid‐ and high‐marsh species occurred at lower elevations, supporting experimental studies showing that species’ lower elevation limits can also be controlled by competition (Bockelmann & Neuhaus, 1999). Intriguingly, while MRs often show significant differences in vegetation from natural marshes (Mossman et al., 2012a; Wolters et al., 2005b), sites restored by encouraging sedimentation through the use of bunds (more closely replicating sedimentary and successional conditions of natural saltmarshes) have developed equivalent vegetation (Van Loon‐Steensma, Van Dobben, Slim, Huiskes, & Dirkse, 2015). This may indicate that availability of bare sediment at high elevations during restoration (Figure S1) leads to marshes restored by MR following an inhibition successional model rather than a facilitation model (Connell & Slatyer, 1977).

Under the inhibition model, priority effects are important; species with good dispersal and establishment pre‐empt bare sediment and exclude others. Thus, differences in dispersal ability, which may limit colonization by some species (Rand, 2000; Wolters, Garbutt, & Bakker, 2005a), may have longer term consequences for vegetation development. The mid‐marsh dominants *Puccinellia* and *Atriplex* occurred more frequently on MRs than the suite of mid‐marsh species deficient on ARs (*Limonium*,* Plantago* and *Triglochin*) so by establishing earlier, the dominant species may be able to inhibit the later establishment of other mid‐marsh species. Seed dispersal and viability of individual species appears variable (Erfanzadeh et al., 2010; Hutchings & Russell, 1989; Wolters et al., 2004), although the greater frequency of *Atriplex* and *Puccinellia* in natural marshes (Mossman et al., 2012a) suggests higher propagule availability of these species. They also have taller growth forms than the species deficient on ARs*,* and have the potential for rapid clonal spread (Table S2), which may give them a competitive advantage. Some species appear to be able to recover from dispersal limitation. In this study, the lower than expected occurrence frequency of *Spartina* in MRs may be due to dispersal limitation, as it has low seed viability (Marks & Truscott, 1985). The effects of this dispersal limitation appear to be short‐term as *Spartina* was more frequent than expected in ARs, likely due to rapid clonal spread.

It is possible that the marsh age effects we observe are due to an environmental characteristic not measured in this study. Restored saltmarshes have harder upper‐marsh sediments (Brooks et al., 2015), which could limit the ability of species to establish high in the tidal frame. Patches of bare sediment, especially at high elevations, can become hypersaline in the summer, impeding colonization (Bertness, Gough, & Shumway, 1992; Srivastava & Jefferies, 1996), although shading by vegetation may reduce this and facilitate the colonization of other species (Bertness & Hacker, 1994). These processes may explain why mid‐ and high‐marsh species did not occur at as high elevations in MRs as in natural marshes (Figure 4). Finally, sediment structure and chemistry in restored marshes carry legacies of former land use (Macleod, Scrimshaw, Emmerson, Chang, & Lester, 1999; Spencer et al., 2017; Tempest, Harvey, & Spencer, 2015). Residual nutrients in former agricultural soils can favour upper‐marsh dominants (Van Wijnen & Bakker, 1999), which would be consistent with mid‐ and high‐marsh species occurring at lower elevations in restored marshes. The magnitude and length of the legacy of former agricultural land use are poorly understood, so further research is needed to evaluate their likely importance for restoration outcomes. If co‐located measurements of these variables and plant communities were available, niche models could be used to evaluate their effects.

### 4.4 Management actions to improve restoration outcomes

Our niche modelling shows that failure to create appropriate environmental conditions is not sufficient to explain all differences in plant occurrence, and that differences in succession are likely to be important. We suggest two specific management actions to benefit underrepresented species on restored marshes. First, our niche models indicate that these species were most likely to occur in areas with low redox potential high in the tidal frame, so are likely to benefit from the creation of poorly drained conditions at high elevations in the marsh, perhaps through the construction of depressions. Second, planting or sowing seeds of these species may help them overcome inhibition from dominant species and establish in restored marshes. At smaller scales, trampling by grazing herbivores might create the required environmental conditions (Schrama et al., 2013) and reduce competitive dominance (Bos, Bakker, de Vries, & van Lieshout, 2002), but would have complex multi‐trophic effects (Davidson et al., 2017).

### 4.5 Using niche models to improve prediction in restoration ecology

Understanding the contribution of environmental suitability and altered succession is a major challenge in predicting restoration outcomes (Brudvig et al., 2017). We show that niche models can make an important contribution to this. Differences in environmental conditions between natural and restored sites partially explain differences in vegetation, so more closely replicating the environment of natural sites should improve restoration outcomes. But discrepancies in plant occurrence are likely to remain, and niche models can help identify the processes responsible. We tested for signatures of altered successional processes by looking at whether species shifted their elevation niches. Initial availability of bare sediment across the tidal frame allowed good dispersers to shift their realized niche, subsequently inhibiting establishment of poorer dispersers—explaining the long‐term underrepresentation and overrepresentation of species. The priority effects and subsequent inhibition–succession identified here are likely to be important in other systems, e.g. Californian grasslands, where the outcome of restoration is influenced by the species favoured to establish in the year of restoration (Stuble, Fick, & Young, 2017).

## AUTHORS’ CONTRIBUTIONS

M.J.P.S. and H.L.M. conceived the study, A.J.D., A.G. and H.L.M. designed the field sampling methodology and collected the data, M.J.P.S. analysed the data with input from H.L.M., A.J.D. and A.G. All authors contributed to writing the manuscript and approved the final version.

## DATA ACCESSIBILITY

Data available from the Dryad Digital Repository https://doi.org/10.5061/dryad.380g2 (Sullivan, Davy, Grant, & Mossman, 2017).
