# Supplementary material for: Is saltmarsh restoration success constrained by matching natural environments or altered succession? A test using niche models
Source: J Appl Ecol. 2017 Nov 22;55(3):1207–17. doi: 10.1111/1365-2664.13033 (PMC5947831; doi:10.1111/1365-2664.13033)
Supplement: Supplementary file 1 [file JPE-55-1207-s001.docx]

**Supporting information for “Is saltmarsh restoration success constrained by matching natural environments or altered succession? A test using niche models”**

Martin J. P. Sullivan^1^, Anthony J. Davy^2^, Alastair Grant^3^, Hannah L. Mossman^4^

^1^ School of Geography, University of Leeds, Leeds, LS2 9JT, UK.

^2^ School of Biological Sciences, University of East Anglia, Norwich Research Park, Norwich, NR4 7TJ, UK.

^3^ School of Environmental Sciences, University of East Anglia, Norwich Research Park, Norwich, NR4 7TJ, UK.

^4^ School of Science and the Environment, Manchester Metropolitan University, Manchester, M1 5GD, UK.

Table S1. Details and locations of all sites used in this study.

Table S2. Life-history traits of study species.

Table S3. Cross-validation performance of niche models for each study species.

Table S4. Environmental limits of study species.

Table S5. Independent effect of redox, relative tidal height and marsh age in determining the occurrence of ten saltmarsh plant species.

Figure S1. Percentage cover of bare ground across the tidal frame.

**Table S1.** Locations and ages of study sites. Age is the age of the marsh when sampled

| Age class | Region | Site | Year of breach | Age | Distance to natural marsh | Lat | Long |
| --- | --- | --- | --- | --- | --- | --- | --- |
| MR | Essex | Abbott's Hall MR | 2002 | 2 | 0.7 | 51.78 | 0.85 |
| MR | Norfolk | Brancaster MR | 2002 | 4 | 0.3 | 52.98 | 0.64 |
| NAT | Essex | Brightlingsea Ref | - | - | - | 51.81 | 1.04 |
| AR | Essex | Barrow Hill AR | 1953 | 52 | 0.5 | 51.81 | 0.93 |
| NAT | Norfolk | Brancaster Sand | - | - | - | 52.97 | 0.62 |
| AR | Essex | Foulton Hall A AR | 1921 | 84 | 1.4 | 51.92 | 1.26 |
| MR | Norfolk | Freiston Shore MR | 2002 | 2 | 0.4 | 52.98 | 0.10 |
| NAT | Norfolk | Freiston Ref | - | - | - | 52.96 | 0.09 |
| AR | Essex | Hemley AR | 1953 | 52 | 0.3 | 52.05 | 1.34 |
| NAT | Essex | Hemley Ref | - | - | - | 52.05 | 1.35 |
| NAT | Norfolk | Titchwell Ref | - | - | - | 52.97 | 0.61 |
| NAT | Essex | Tollesbury Ref | - | - | - | 51.77 | 0.84 |
| MR | Humber | Paull Holme Strays MR | 2003 | 2 | 1.8 | 53.71 | -0.22 |
| NAT | Norfolk | Thornham Ref | - | - | - | 52.97 | 0.58 |
| AR | Norfolk | Titchwell AR | 1953 | 51 | 0.6 | 52.97 | 0.62 |
| MR | Essex | Tollesbury MR | 1995 | 9 | 0.3 | 51.77 | 0.84 |
| NAT | Essex | Wallasea Island Ref | - | - | - | 51.61 | 0.82 |
| MR | Essex | Wallasea MR | 2006 | 4 | 0.5 | 51.62 | 0.83 |
| NAT | Norfolk | Wells | - | - | - | 52.96 | 0.90 |
| NAT | Essex | Steeple Ref | - | - | - | 51.71 | 0.80 |
| NAT | Norfolk | Wareham Ref | - | - | - | 52.97 | 0.89 |
| MR | Norfolk | Freiston Shore MR | 2002 | 2 | 0.4 | 52.98 | 0.10 |

**Table S2.** Life-history traits of study species. Data from PLANTATT (Hill, Preston & Roy 2004) unless otherwise stated.

| Species | Height (cm) | Perennial/ Annual | Life-form | Woody growth | Clonal growth |
| --- | --- | --- | --- | --- | --- |
| *Atriplex portulacoides* | 80 | Perennial | Nanophanerophyte | y | Long node* |
| *Aster tripolium* | 100 | Perennial | Hemicryptophyte | n | None |
| *Elytrigia atherica* | 105 | Perennial | Hemicryptophyte | n | Long rhizome |
| *Limonium vulgare* | 40 | Perennial | Hemicryptophyte | n | Short rhizome ** |
| *Plantago maritima* | 15 | Perennial | Hemicryptophyte | n | None |
| *Puccinellia maritima* | 80 | Perennial | Hemicryptophyte | n | Long rhizome |
| *Salicornia europaea agg.* | 38 | Annual | Therophyte | n | None |
| *Spartina anglica* | 130 | Perennial | Hemicryptophyte | n | Long rhizome |
| *Suaeda maritima* | 30 | Annual | Therophyte | n | None |
| *Triglochin maritimum* | 55 | Perennial | Hemicryptophyte | n | Short rhizome |

* Classed as none from PLANTATT, but updated from personal observation supported by (Chapman 1950)

** Classed as none from PLANTATT, but updated from personal observation supported by (Boorman 1967; Boorman 1968)

**Table S3.** Performance of niche models for each study species at classifying the suitability of quadrats. Model performance was quantified using the area under the receiver operating characteristic curve (AUC). Models were trained on a subset (75 %) of data from natural marshes, and tested on an independent subset (25 %) of natural marsh data. This was repeated 1000 times. The median and 95 % CIs of from these evaluations are shown. Note that AUC values tend to be higher for species that occur infrequently, so the relative occurrence area (≡ frequency of occurrence in quadrats in natural marshes) is shown.

| Species | AUC (median, 95% CIs in parenthesis) | Relative occurrence area |
| --- | --- | --- |
| *Spartina anglica* | 0.75 (0.65 – 0.85) | 0.16 |
| *Salicornia europaea* | 0.73 (0.65 – 0.81) | 0.42 |
| *Suaeda maritima* | 0.61 (0.52 – 0.74) | 0.48 |
| *Aster tripolium* | 0.66 (0.57 – 0.74) | 0.38 |
| *Puccinellia maritima* | 0.77 (0.69 – 0.84) | 0.69 |
| *Atriplex portulacoides* | 0.74 (0.66 – 0.81) | 0.56 |
| *Limonium vulgare* | 0.77 (0.69 – 0.83) | 0.44 |
| *Triglochin maritima* | 0.76 (0.68 – 0.84) | 0.23 |
| *Plantago maritima* | 0.74 (0.61 – 0.83) | 0.09 |
| *Elytrigia atherica* | 0.91 (0.85 – 0.97) | 0.07 |

**Table S4.** Environmental limits of study species on natural marshes (*n* = 506 quadrats). The minimum, median, maximum and range of relative tidal height and redox potential values of quadrats each species was recorded in are shown.

| Species | Relative tidal height | | | | Redox potential (mV) | | | |
| --- | --- | --- | --- | --- | --- | --- | --- | --- |
|  | Minimum | Median | Maximum | Range | Minimum | Median | Max | Range |
| *Spartina anglica* | -0.36 | 0.59 | 1.01 | 1.37 | -237 | 233 | 414 | 651 |
| *Salicornia europaea* | -0.43 | 0.68 | 1.63 | 2.06 | -331 | 275 | 469 | 800 |
| *Suaeda maritima* | -0.12 | 0.75 | 1.25 | 1.37 | -331 | 309 | 469 | 800 |
| *Aster tripolium* | 0.13 | 0.77 | 1.27 | 1.14 | -46 | 308 | 438 | 484 |
| *Puccinellia maritima* | 0.18 | 0.80 | 1.27 | 1.09 | -72 | 309 | 438 | 510 |
| *Atriplex portulacoides* | 0.31 | 0.83 | 1.27 | 0.96 | 30 | 332 | 446 | 416 |
| *Limonium vulgare* | 0.27 | 0.85 | 1.25 | 0.98 | -46 | 304 | 417 | 463 |
| *Triglochin maritima* | 0.40 | 0.85 | 1.25 | 0.85 | 30 | 294 | 387 | 357 |
| *Plantago maritima* | 0.56 | 0.89 | 1.23 | 0.67 | 164 | 306 | 390 | 226 |
| *Elytrigia atherica* | 0.74 | 1.00 | 1.38 | 0.64 | 224 | 368 | 446 | 222 |

**Table S5.** Independent effect (%) of redox, relative tidal height and marsh age in determining the occurrence of ten saltmarsh plant species. The occurrence of each species was modelled using binomial generalized linear models, with the independent effect determined using hierarchical partitioning (Walsh & Mac Nally 2013). Region was included as an explanatory variable in all models. *n* = 1044 quadrats.

| Species | Redox potential | Relative tidal height | Marsh age (MR, AR or NAT) |
| --- | --- | --- | --- |
| *Spartina anglica* | 42 | 22 | 38 |
| *Salicornia europaea* agg. | 43 | 28 | 29 |
| *Suaeda maritima* | 13 | 52 | 35 |
| *Aster tripolium* | 51 | 40 | 9 |
| *Puccinellia maritima* | 26 | 49 | 25 |
| *Atriplex portulacoides* | 52 | 31 | 17 |
| *Limonium vulgare* | 17 | 39 | 44 |
| *Triglochin maritima* | 22 | 21 | 57 |
| *Plantago maritima* | 16 | 41 | 43 |
| *Elytrigia atherica* | 48 | 49 | 4 |


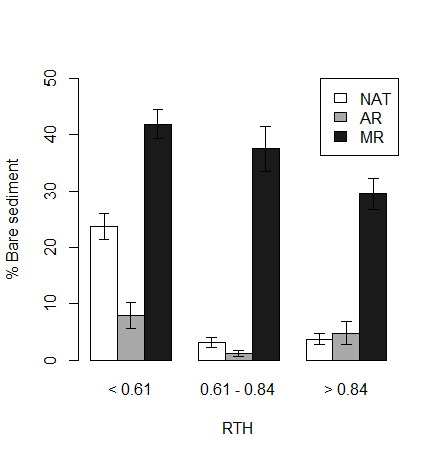


Figure S1. Percentage cover of bare ground across the tidal frame. Mean values for each marsh age are shown for the lowest (RTH < 0.61), middle (RTH 0.61 – 0.84) and highest (RTH > 0.84) thirds of quadrats. Error bars show standard errors.

Additional references

Boorman, L.A. (1967) L*imonium vulgare M*ill. and L*. humile M*ill. *Journal of Ecology,* **55,** 221-232.

Boorman, L.A. (1968) Some aspects of the reproductive biology of L*imonium vulgare M*ill., and L*imonium humile M*ill. *Annals of Botany,* **32,** 803-824.

Chapman, V. (1950) Halimione portulacoides (L.) Aell. *Journal of Ecology,* **38,** 214-222.

Hill, M.O., Preston, C.D. & Roy, D.B. (2004) *PLANTATT-attributes of British and Irish plants: status, size, life history, geography and habitats*. Centre for Ecology & Hydrology, Abbotts Ripton, UK.

Walsh, C. & Mac Nally, R. (2013) hier.part: Hierarchical Partitioning. R package version 1.0-4. <http://CRAN.R-project.org/package=hier.part>.
